# The Intersection of m6A Methylation and Immune Response in PCOS: A Bioinformatics Perspective

**DOI:** 10.1002/iid3.70376

**Published:** 2026-02-22

**Authors:** Wenting Xu, Lingli Shi, Aifang Lu, Lijuan Cui, Haiqing Qian, Jiahui Wang, Mengyu Tang, Lili Zhu, Lihong Wang

**Affiliations:** ^1^ State Key Laboratory on Technologies for Chinese Medicine Pharmaceutical Process Control and Intelligent Manufacture, Zhangjiagang TCM Hospital Affiliated to Nanjing University of Chinese Medicine Nanjing China; ^2^ Zheng's Gynecology Department Kunshan Traditional Chinese Medicine Hospital Suzhou Jiangsu China; ^3^ Department of Pathology Zhangjiagang TCM Hospital Affiliated to Nanjing University of Chinese Medicine Suzhou China; ^4^ Department of Gynecology Zhangjiagang TCM Hospital Affiliated to Nanjing University of Chinese Medicine Suzhou China

**Keywords:** bioinformatics, immune infiltration, methylation, polycystic ovary syndrome

## Abstract

**Background:**

Polycystic ovary syndrome (PCOS) is a prevalent endocrine disorder, the molecular underpinnings of which remain largely undefined. The most common methylation modification of RNA, *N* ^6^‐methyladenosine (m6A), plays an important role in various reproductive and endocrine disorders. This study investigates key m6A genes in PCOS and their association with immune cell infiltration using advanced bioinformatics methods.

**Methods:**

We utilized gene expression data and clinical information from the Gene Expression Omnibus database data sets GSE137684, GSE80432, and GSE114419. The expression of m6A‐related genes was analyzed across all samples. Using the GSVA and CIBERSORT packages in R, we developed a diagnostic model based on the m6A gene–protein interaction network, conducted enrichment analysis of hub genes, and assessed the correlation between these genes and immune cell infiltration.

**Results:**

Analysis of data sets GSE137684 and GSE804322 identified variable expression patterns among three categories of m6A genes. A diagnostic model centered on m6A gene expression was established, highlighting five genes—WTAP, METTL14, ZC3H13, PCIF1, and RBM15—with significant effect coefficients. Unsupervised clustering of hub genes indicated that METTL14, HNRNPA2B1, YTHDF3, YTHDF2, YTHDC1, and YTHDC2 are potential discriminators in PCOS. The analysis of immune infiltration revealed a correlation between m6A regulators and immune cell levels, with METTL3 showing the most significant regulatory impact.

**Conclusion:**

*N* ^6^‐methyladenosine RNA methylation regulators are intricately linked with the development of PCOS and may influence immune cell infiltration in affected individuals. This study enhances our understanding of the molecular interactions in PCOS and suggests potential biomarkers for diagnosis and targets for therapeutic intervention.

## Graphical abstract

*N* ^6^‐methyladenosine RNA methylation regulators are intricately linked with the development of polycystic ovary syndrome (PCOS) and may influence immune cell infiltration in affected individuals. This study enhances our understanding of the molecular interactions in PCOS and suggests potential biomarkers for diagnosis and targets for therapeutic intervention

**Abbreviations**
AUC: area under the curve
CD8: cluster of differentiation 8
DCA: decision curve analysis
FTO: fat mass and obesity‐associated protein
IR: insulin resistance
m6A: *N* ^6^‐methyladenosine
NES: normalized enrichment score; NIH, National Institutes of Health
PCA: principal component analysis
PCOS: polycystic ovary syndrome
PPIN: protein–protein interaction network
PR: precision–recall curve
ROC curve: receiver operating characteristic curve
TFs: transcription factors

## 1 Background

Polycystic ovary syndrome (PCOS) is a complex female reproductive dysfunction and metabolic disorder characterized by continuous anovulation, insulin resistance (IR), and excessive androgen production. Approximately 10%–15% of women of childbearing age worldwide are affected by PCOS. Long‐term metabolic disorders in PCOS patients increase the incidence of diabetes, cardiovascular disease, and endometrial cancer in the long term, seriously affecting the health of women. Therefore, a further understanding of the etiology of PCOS is important for its diagnosis, treatment, and prevention.

The cause of PCOS is complicated, and there is no ethnic difference, which is the result of various factors, such as genetics, environment, and lifestyle. Low progesterone and high androgen status in PCOS patients can cause abnormal activation of the immune system, leading to the production of various inflammatory factors and autoantibodies in patients, generating a chronic inflammatory state in the body. In recent years, the role of immune dysfunction in the occurrence of PCOS has gradually attracted attention. Studies have shown that more lymphocytes and macrophages are infiltrated in ovarian tissues of PCOS patients than in non‐PCOS women, and various cell factors, such as inflammatory factors, granulocyte–macrophage colony‐stimulating factor, and insulin growth factor‐1, can lead to ovulation disorder [1].

At present, there are many studies on the genetic etiology of PCOS, and some results show that epigenetics plays a certain role in the occurrence and development of the disease. Epigenetic regulation is a ubiquitous regulatory mechanism in life processes that plays a decisive role in stem cell maintenance, self‐renewal and differentiation, individual ageing and developmental abnormalities, and the occurrence and development of diseases.

The study of epigenetics has been increasingly widely recognized in the academic field and has become an important frontier and area of attention in the field of life science. Epigenetic mechanisms in PCOS are a current research hotspot. However, due to the various types of epigenetics and their different roles in the occurrence of PCOS, there are still many unsolved mysteries about its specific mechanism.

The methylation of *N* ^6^‐methyladenosine (m6A) of eukaryotic mRNA, found in 1974, is the most common posttranscriptional modification of mRNA, accounting for 80%. Posttranscriptional regulation occurs through m6A modification in mammalian cells and is dynamically reversible. As well as regulating gene expression, splicing, RNA editing, and RNA stability, it also controls mRNA longevity and degradation, and mediates translation of ceRNA. Despite the importance of m6A methylation modification in immune‐related biological processes (BPs), the integrated landscape of immune cell infiltration features mediated by m6A methylation modification in PCOS remains unclear.

In this study, the differential expression of m6A RNA methylation regulators was analyzed using RNA sequencing data from the Gene Expression Omnibus (GEO) data set. In addition, we further analyzed the relevance of m6A RNA methylation to immune cell infiltration features in PCOS.

## 2 Methods

### 2.1 Data and Preprocessing

Gene expression profiles and clinical metadata were retrieved from the GEO database (https://www.ncbi.nlm.nih.gov/geo). GEO is an internationally public, authoritative, and extensively used gene expression data repository, containing a large volume of transcriptomic and microarray data that has undergone rigorous quality control, aligning with National Institutes of Health (NIH)‐maintained standardized quality control protocols [2]. For this study focusing on human ovarian granulosa cells (GCs) in PCOS, the availability of public expression profiling data is limited. The data sets selected from GEO for this research (GSE137684, GSE80432, and GSE114419) are not only directly relevant to human ovarian GCs but also possess complete clinical grouping information, thereby satisfying the requirements for sample consistency and data integrity. Furthermore, GEO's data sets were found to be superior in terms of sample origin, sequencing platform consistency, and data accessibility when compared with other databases. This selection ensures a sufficient data foundation for subsequent integrated analysis, batch effect correction, and model validation, thus guaranteeing the scientific rigor and reproducibility of the study.

Data on gene expression from GSE137684 and GSE80432 data sets as well as clinical information related to the samples were downloaded from the GEO database (https://www.ncbi.nlm.nih.gov/geo). Sequencing platforms used were GPL17077 and GPL6244, and the samples were all human ovarian GCs. In data set GSE137684, there are 12 samples, including 4 from controls and 8 from PCOS patients; in data set GSE80432, there are eight samples, including four from controls and four from PCOS patients. The above two data sets were integrated for downstream analysis, the batch effects between different data sets were corrected and log 2 normalized using the R package sva [3], and the standardized and batch‐corrected expression distributions were visualized using box plots.

The preprocessed data set GSE114419 from the GEO database was used for the external validation of the diagnostic model. The samples of this data set are human ovarian GCs, and the sequencing platform is GPL17586. This data set has six samples, including three control samples and three PCOS patient samples.

### 2.2 Construction of a Panoramic Map of the m6A Genes

To analyze the expression levels of the m6A genes in all samples, the m6A‐related genes were first obtained from [4, 5, 6, 7]; the genes included 11 writer genes (METTL3, METTL14, WTAP, VIRMA, ZC3H13, CBLL1, RBM15, RBM15B, METTL16, ZCCHC4, and PCIF1), 26 reader genes (YTHDF1, YTHDF2, YTHDF3, YTHDC1, YTHDC2, YTHDC3, HNRNPA2B1, HNRNPC, RBMX, IGF2BP1, IGF2BP2, IGF2BP3, FMR1, PRRC2A, EIF3A, EIF3B, EIF3H, LRPPRC, SRSF3, NXF1, TRMT112, NUDT21, CPSF6, SETD2, SRSF10, and X3RN1), and three eraser genes Fat mass and obesity‐associated protein (FTO, ALKBH5, and ALKBH3), for a total of 40 genes. After comparison with the existing expression profiles, 36 genes were retained (excluding VIRMA, YTHDC3, HNRNPC, and RBMX).

The expression heatmap of these genes in all samples was first plotted using the R package pheatmap [8], and then the box plots of the two groups (the control and patient groups) were plotted by the R package ggpubr [9]. This study used RCircos [10] to map 36 genes' chromosomal localization, Chromosome data were provided by this package, and information on the positions of the genes on chromosomes was obtained from ENSEMBL [11].

### 2.3 Correlation Analysis Between Writer and Eraser Genes

Further analysis of any two genes' expression levels was performed by calculating the Pearson correlation coefficient between them, and it was evident that a correlation existed when the absolute correlation coefficient exceeded 0.7 and the *p* value was less than 0.01. The R package ggplot2 [12] was used to generate a scatter plot of the correlations between the gene pairs that met the criteria and to fit the correlation curve, and the R package ggExtra [13] was used to plot the histogram and density curve, which are outside of the graph.

### 2.4 Construction of an m6A Gene‐Based Diagnostic Model

It is possible for normal samples and patient samples to have different levels of m6A modification due to the important influence of m6A modification. Therefore, it is extremely valuable to construct an m6A gene‐based diagnostic model.

First, ridge regression was performed to screen all m6A genes using the R package glmnet [14], and the optimal lambda value was selected. After ridge regression was performed, only genes with a nonzero coefficient were retained. Subsequently, further gene screening was carried out using logistic regression, and the genes and coefficients of the model were plotted on a forest plot using the R package forestplot [15].

Subsequently, based on the nomogram, visualizing the clinical significance of the model was achieved using the R package rms [16]. With the R package pROC [17], the receiver operating characteristic (ROC) curve of the diagnostic model was plotted and the area under the ROC curve (AUC) was calculated. At the same time, to illustrate the effectiveness of the nomogram, precision–recall (PR) and decision curve analysis (DCA) curves were plotted with the external data set GSE114419 for verification. With the R package ggDCA, DCA curves were plotted [18].

### 2.5 Construction of the Protein–Protein Interaction Network (PPIN)

There is often a strong correlation between the expression levels of different genes, particularly genes that regulate the same BP. Hence, a PPIN was created using m6A‐associated genes to uncover their relationship.

In the STRING database [19], the default confidence threshold of 0.4 was used to construct the PPIN. Subsequently, the PPIN was exported using Cytoscape software [20] for further analysis. Each node's network characteristics were computed, utilizing the Cytohubba plug‐in [21] to identify hub nodes based on node degree, with the top 10 nodes by degree designated as hub nodes. Considering hub nodes are often highly correlated with other nodes, they may play an extremely important role in regulating the entire BP.

Thus, we performed prediction studies on 10 hub nodes based on the miRNet database [22], that is, the prediction of microRNAs (miRNAs) and transcription factors (TFs). A plot was created using Cytoscape after the prediction results were exported.

### 2.6 Unsupervised Clustering of Samples

To resolve the heterogeneity between patients, unsupervised clustering can be performed on samples based on the hub genes.

The optimal number of clusters was determined using the R package factoextra [23], the unsupervised clustering of all patients was performed using *k*‐means clustering, and two clusters were finally obtained. Similarly, the final clustering results were shown using this R package. On the basis of the expression levels of the 10 hub genes in both samples groups, a heatmap was created. The R package ggpubr [9] was used to plot the combined box plot and violin plot according to the clusters of the samples.

### 2.7 Gene Enrichment Analysis

Following data preprocessing, which included normalization and batch effect correction using the R package sva [3], gene differential expression analysis was performed to further reveal the biological differences between the two sample groups. Genes with a corrected *p* value less than 0.05 and an absolute log 2 fold change (FC) value greater than 1 were considered differentially expressed genes (DEGs). The results of this analysis are displayed in volcano plots and heatmaps. The volcano plot was utilized to intuitively display the significance and expression change trends of all genes, facilitating the rapid identification of upregulated and downregulated genes. The heatmap illustrates the expression patterns of these DEGs across the various samples, aiding in the visualization of differences between groups. These identified DEGs were subsequently used for gene set enrichment analysis (GSEA).

Enrichment analysis by Gene Ontology (GO) is one of the most common approaches for analyzing functional enrichment in different dimensions and levels of genes. GO enrichment analysis is generally performed at three levels, that is, molecular function, BP, and cellular component [24]. Kyoto Encyclopedia of Genes and Genomes (KEGG) stores information about genomes, diseases, and drug targets [25]. Using the R package clusterProfiler [26], GO functional annotation and KEGG pathway enrichment of all DEGs were performed to identify significantly enriched BPs. Bar and bubble graphs were used to visualize enrichment results. A significance threshold of 0.05 was applied to the enrichment analysis.

GSEA is a computational method used to determine whether there are significant differences in a set of predefined genes between two biological states, and it is typically used to estimate changes in the activities of pathways and BPs in gene expression data sets [27]. To examine variances in BPs between the two sample groups based on the gene expression profile data set, the reference gene sets “c5.go.v7.4.entrez.gmt” and “c2.cp.kegg.v7.4.entrez.gmt” were obtained from the MSigDB database [28], and the GSEA method included in the R package “clusterProfiler” was used for enrichment analysis and visualization. A corrected *p* value of < 0.05 was considered statistically significant.

### 2.8 Immune Infiltration Analysis

The immune microenvironment is mainly composed of immune cells, inflammatory cells, fibroblasts, interstitial tissues, and various cytokines and chemokines, and it is a complex integrated system of loads. Infiltration analyses of immune cells in tissues have an important guiding role in disease research and prognosis prediction.

To further explore the similarities and differences in immune cell infiltration levels between the two groups of samples, the ssGSEA‐based method was performed using the R package GSVA [29]. Twenty‐eight marker genes for immune cells were obtained from [30]. and used as a background gene set to perform ssGSEA for each sample. The infiltration of all immune cells is displayed in heatmaps and box plots.

To maximize the accuracy of the research results, the R package CIBERSORT [30] was used to assess the immune cell infiltration level through a different method, and based on the LM22 background gene set provided by CIBERSORT, the contents of 22 types of immune cells in each sample were calculated to reflect the infiltration level. The results are presented in stacked histograms and box plots.

In addition, to directly examine the correlations between the hub genes and immune cell infiltration, correlation scatter plots were generated, and correlation curves were fitted for gene‐immune cell pairs with significant correlations. Additionally, histograms and density curves were plotted outside of the graph using the R package ggExtra [13].

## 3 Results

### 3.1 Panoramic Map of the m6A Genes

**Figure 1:**
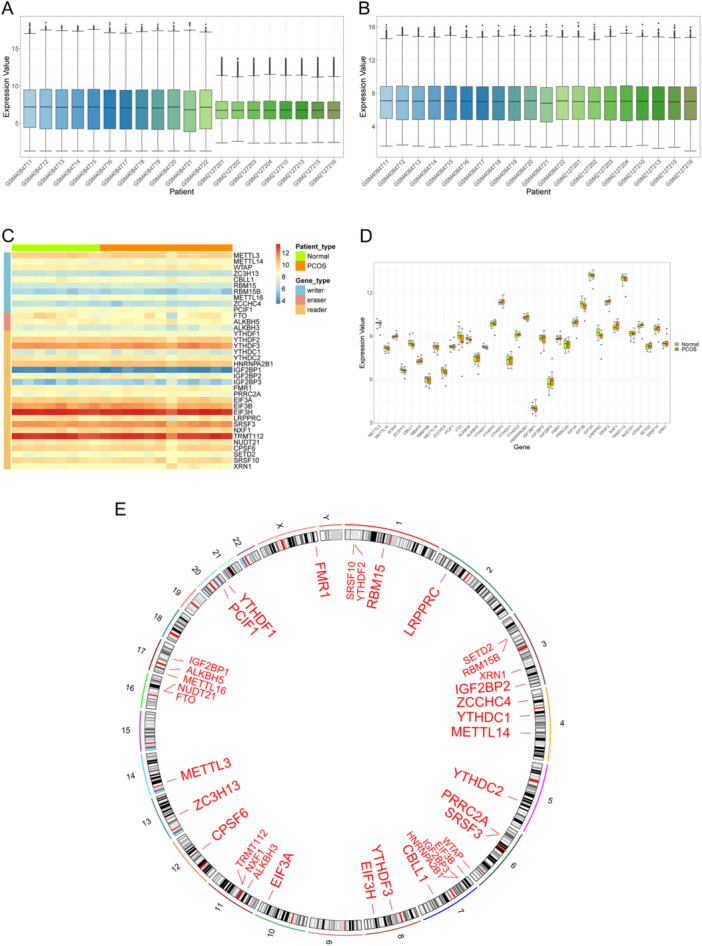
Data preprocessing and m6A gene panoramic map. (A) The expression distribution after log normalization without batch effect correction. The *x*‐axis is the sample, and the *y*‐axis is the gene expression level. The line in the box plot is the median, the upper box line is the upper quartile, the lower box line is the lower quartile, the upper and lower boxes are the limits, and the dots are the outlier data. (B) The expression distribution after batch effect correction and log normalization. (C) Heatmap of m6A gene expression. Behavioral genes are listed as samples. Samples and different types of genes are labeled with different color bands. (D) Box plot of m6A gene expression. The *x*‐axis is the gene, the *y*‐axis is the gene expression level, and the sample groups are distinguished by different colors. (E) Circle map of the location of the gene on the chromosome. The outer circle is the chromosome, and the inner circle is the location of the gene on the chromosome. m6A, *N* ^6^‐methyladenosine; PCOS, polycystic ovary syndrome.

A panoramic map of m6A‐related genes across all samples was constructed by integrating expression profiles from the GSE137684 and GSE80432 data sets. To address batch effects and normalize raw data, batch effect correction and logarithmic normalization were applied to data from diverse sources. As shown in Figure 1A,B, the expression distributions across all samples were consistent following batch correction and normalization, thereby enhancing the accuracy and robustness of downstream analyses.

The expression levels of all m6A genes in normal and patient samples were visualized using heatmaps and grouped box plots (Figure 1C,D). Differential expression was observed among the three m6A functional types. For instance, the writer gene CBLL1 and the eraser gene FTO were downregulated in PCOS patients, whereas the reader gene NXF1 was upregulated. In addition, the chromosomal localization of these genes was obtained to plot a panoramic map (Figure 1E). According to the results, the chromosomal locations of some genes were very close, suggesting that they are closely related at the genomic level and might also have similar transcriptome characteristics.

### 3.2 Correlations Between Writer and Eraser Genes

**Figure 2:**
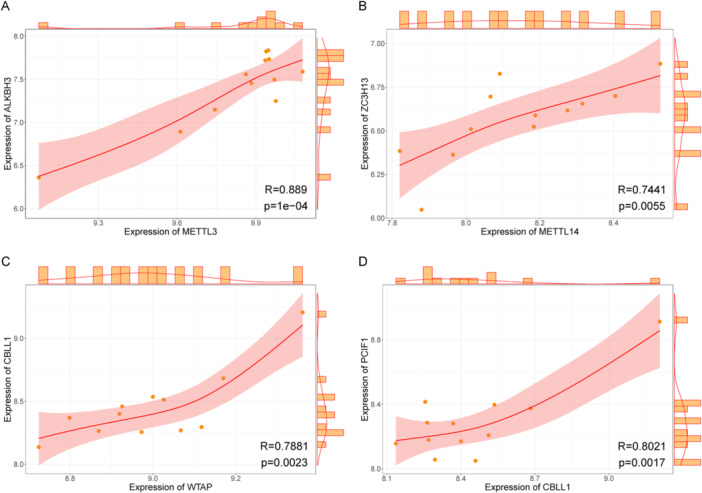
Correlation between writer and eraser genes. Each dot in the figure represents a patient sample. The curve is the correlation fitting curve, the shaded area is the confidence interval, and the histogram and the density curve are outside of the graph. (A) METTL3‐ALKBH3 correlation, (B) METTL14‐ZC3H13 correlation, (C) WTAP‐CBLL1 correlation, and (D) CBLL1‐PCIF1 correlation.

Correlation analyses between writer and eraser genes were performed by calculating correlation coefficients, generating scatter plots, and fitting correlation curves. Four gene pairs, including METTL3‐ALKBH3, METTL14‐ZC3H13, WTAP‐CBLL1, and CBLL1‐PCIF1 (Figure 2), exhibited statistically significant correlations. All pairs demonstrated strong positive correlations, with coefficients exceeding 0.7. A majority of these genes are writers and erasers, and the writer genes perform similar functions. Therefore, writer gene pairs, such as CBLL1‐PCIF1, may have synergistic effects on expression. The positive correlation between writer and eraser gene pairs (e.g., METTL3‐ALKBH3) may indicate a negative feedback regulatory mechanism between the two or the existence of unknown key pathogenic mechanisms.

### 3.3 The m6A Gene‐Based Diagnosis Model

**Figure 3:**
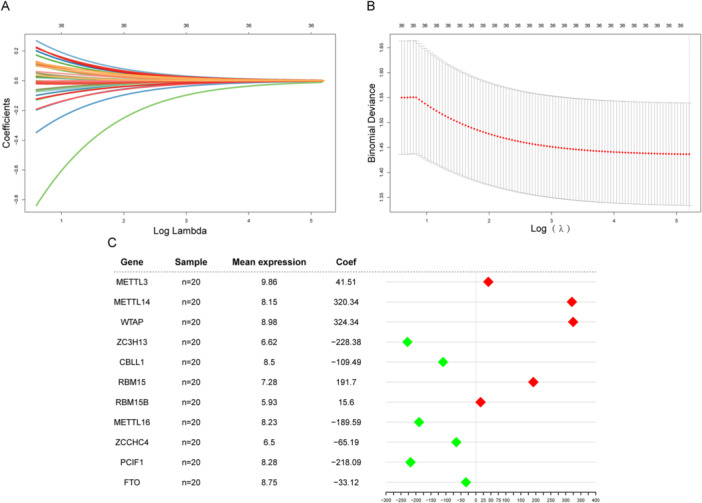
Construction of an m6A gene‐based diagnostic model. (A) Ridge regression curve. This figure shows the convergent screening process of the 36 genes by ridge regression. The *x*‐axis is the log lambda, the *y*‐axis is the regression coefficient, and the lines with different colors represent different features. (B) Lambda value selection curve. This figure is used to select the optimal lambda value of the regression model, and the lowest point is usually selected, that is, the optimal lambda value is at the dotted line in the figure. (C) Forest plot of the diagnostic model. The first column is the 11 genes used in the model, the second column is the number of samples, the third column is the average expression levels of these genes, and the fourth column and the corresponding graph are the influence coefficients of these genes in the model. m6A, *N* ^6^‐methyladenosine.

A diagnostic model for PCOS was developed based on the expression profiles of m6A genes, given their biological significance. Initially, ridge regression was applied to screen 36 m6A genes, and all genes were retained based on optimal lambda values (Figure 3A,B). As a result, 11 genes were retained after a second screening using logistic regression. A forest plot was then employed to visualize the diagnostic model derived from the 11 m6A genes (Figure 3C). The model identified the top five genes with the highest absolute influence coefficients: WTAP (324.34), METTL14 (320.34), ZC3H13 (−228.38), PCIF1 (−218.09), and RBM15 (191.7).

**Figure 4:**
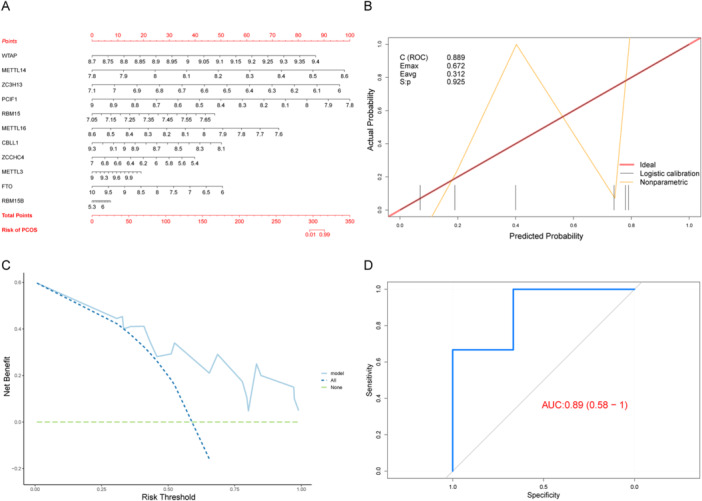
Validation of the m6A gene‐based diagnosis model. (A) Nomogram, with predictive indicators on the left and scales on the right. (B) PR curve. *E*_max_ is the maximum deviation between the model and the ideal model, and *E*_avg_ is the average deviation between the model and the ideal model. *S*:*p*, when *S*:*p* > 0.05, the calibration test is satisfied. *C*(ROC) is the area under the ROC curve. (C) DCA curve. The *x*‐axis is the risk threshold, the *y*‐axis is the net benefit rate, the green dashed line represents the 0 net benefit rate, all (dark blue dashed lines) indicate that all samples are under the intervention, and the model (light blue solid line) indicates the model curve. (D) ROC curve of the model. The *x*‐axis is the specificity, the *y*‐axis is the sensitivity, and the AUC is the area under the curve. DCA, decision curve analysis; m6A, *N* ^6^‐methyladenosine; PR, precision–recall; ROC, receiver operating characteristic.

Subsequently, the nomogram (Figure 4A) was used for visualization to verify the clinical significance of the model. According to the results, most genes influenced the model in the same way, which highlights its accuracy. Next, the PR curve, ROC curve, and DCA curve were used in combination with the external data set to further verify the prediction performance of the model (Figure 4B–D). The results showed that in the three verification methods, the model exhibited superior prediction performance and robustness. ROC and PR curves showed that the overall AUC of this model was 0.89, which indicates excellent diagnostic and predictive capabilities.

### 3.4 PPIN of the m6A Genes

**Figure 5:**
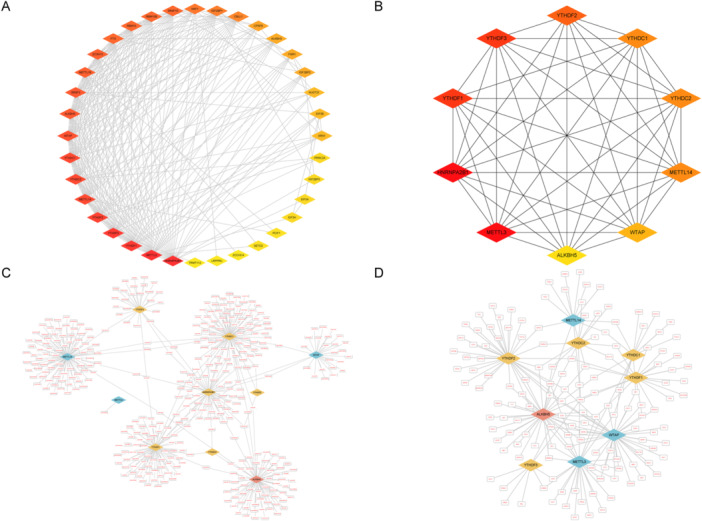
(A) PPIN of 36 m6A genes. The node color reflects the degree, the higher the degree, and the closer the color of the node to the red gradient. (B) Ten hub genes. For the subnetworks of the 10 hub genes extracted from the PPIN, the closer the color of the node to the red gradient, the greater the degree of the node in the original network. (C) miRNA prediction network of the hub genes. The red hollow nodes are miRNAs, and the other nodes represent the hub genes, with the color corresponding to Figure 2C. (D) TF prediction network of the hub genes. Red hollow nodes are TFs, and other colors are the same as (C). m6A, *N* ^6^‐methyladenosine; miRNA, microRNA; PPIN, protein–protein interaction network; TFs, transcription factors.

To investigate the interactions among m6A‐regulating genes, a PPIN was constructed using the STRING database, and visualization was performed with Cytoscape software (Figure 5A). Genes regulating the same BPs often exhibit closer relationships, which provides insights into their functional connections.

The network contained a small number of high‐degree nodes, indicating strong correlations with other nodes. Such nodes often play critical roles within the network. On the basis of degree analysis, the top 10 hub genes were identified (Figure 5B). For a more comprehensive analysis of their regulatory network and genetic background (Figure 5C,D), miRNAs and TFs were predicted using the miRNet database. It appears that these genes have both exclusive and shared miRNAs or TFs, suggesting that they are regulated similarly and perform similar biological functions.

### 3.5 Unsupervised Clustering of the Hub Genes

**Figure 6:**
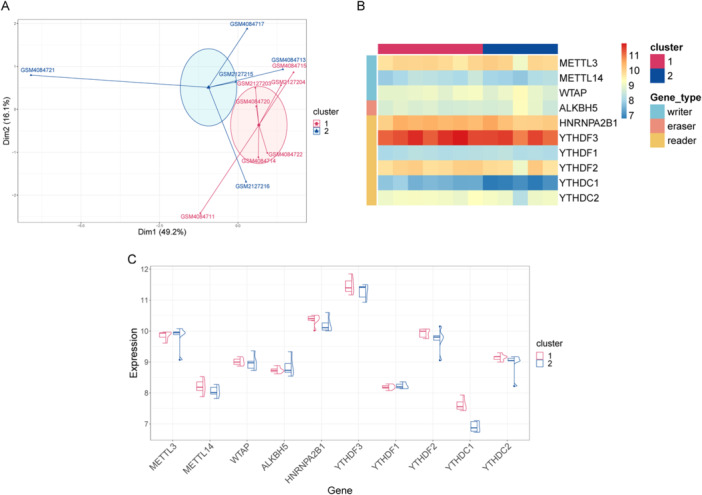
Unsupervised clustering of samples. (A) Dimensionality reduction plot of *k*‐means clustering results. The *x*‐ and *y*‐axes represent two dimensions. Each dot in the figure represents a patient sample. These patients were grouped into two groups, and each color represents a patient subgroup. (B) Heatmap. Behavioral hub genes are listed as samples. The groups and gene types of samples are marked with different color bands. (C) The combined box plot and violin plot. The *x*‐axis is the gene, and the *y*‐axis is the gene expression level. The sample groups are distinguished by different colors. The left half is the box plot, and the right half is the density curve (violin plot).

Hub genes enable the distinction of samples with varying disease states. Using the *k*‐means clustering method based on the expression profiles of 10 hub genes, unsupervised clustering identified two distinct clusters among patient samples. Subsequently, the clustering results were visualized by principal component analysis–based downscaling (Figure 6A), and the results showed that the two groups of samples were clearly classified and that the clustering was excellent.

Figure 6B,C presents a heatmap and a combined box plot with violin plots, illustrating the similarities and differences in hub gene expression levels across the two sample clusters. Significant expression differences were observed between the two groups for METTL14, HNRNPA2B1, YTHDF3, YTHDF2, YTHDC1, and YTHDC2, suggesting that these six genes may serve as critical distinguishing factors. Clustering results are valid and accurate based on this finding.

### 3.6 Biological Differences Between Sample Groups

**Figure 7:**
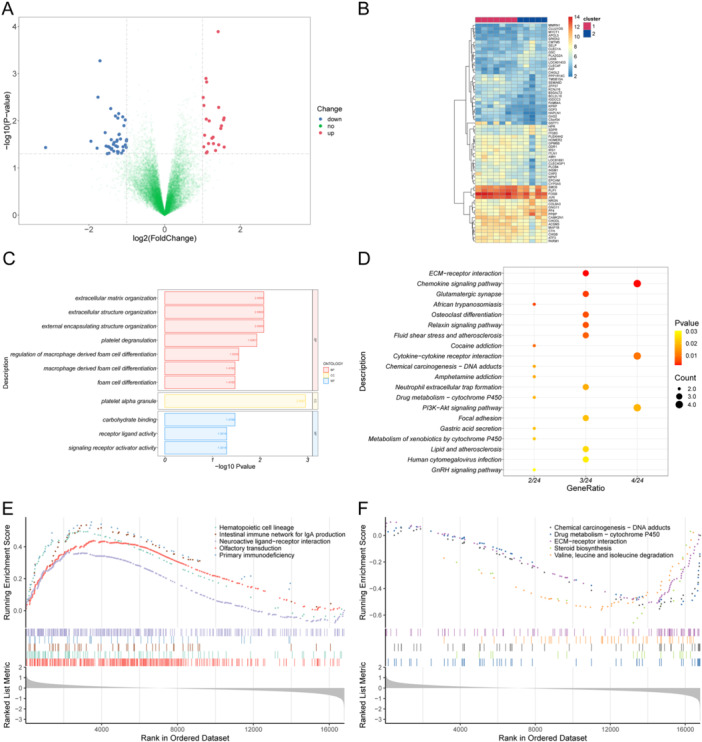
Biological differences between the sample groups. (A) Volcano map of DEGs. The *x*‐axis is log 2 fold change (FC), and the *y*‐axis is −log 10 *p* value. Each dot represents a gene: blue dots represent downregulated genes, red dots represent upregulated genes, and the green dot represents a gene with no significant change in expression. (B) Heatmap of DEGs. The upper color band represents two groups of samples, and each color represents a subgroup. (C) Bar graph of GO enrichment results. The *x*‐axis is −log 10 *p* value, and the *y*‐axis is the enriched GO term. (D) Bubble plot of KEGG enrichment results. The *x*‐axis is the ratio of genes, that is, the number of DEGs that are enriched for the term/the total number of enriched DEGs, and the *y*‐axis is the name of the KEGG pathway. The size of the dot represents the number of genes that are enriched for the term, and the color represents the *p* value. Only the top 20 most significant pathways are shown. (E) GSEA results (upregulation part). The *x*‐axis is the rank of the gene in the DEG list, with upregulation > 0 and downregulation < 0. The upper *y*‐axis is the enrichment score, and the lower *y*‐axis is the log FC value. Each color represents a pathway. Only the top five pathways with the most significant upregulation are shown. (F) GSEA results (downregulation part). Only the top five pathways with the most significant downregulation are shown. DEGs, differentially expressed genes; GO, Gene Ontology; GSEA, gene set enrichment analysis; KEGG, Kyoto Encyclopedia of Genes and Genomes.

Gene differential expression analysis was conducted to identify biological differences between the two sample groups, resulting in the identification of 65 genes meeting the statistical significance threshold. DEGs are visualized in volcano plots and heatmaps (Figure 7A,B).

Following this, GO, KEGG, and GSEA enrichment analyses were conducted to investigate which BPs were affected by these DEGs (Figure 7C–F and Tables 1, 2, 3).

**Table 1:** GO enrichment results of differentially expressed genes (DEGs).

Ontology	ID	Description	GeneRatio	BgRatio	*p* Value	p.adjust	*q* Value	Gene ID	Count
CC	GO:0031091	Platelet alpha granule	5/58	91/19,520	7.43E − 06	0.001099717	0.001008987	PF4/ITGB3/SELP/MMRN1/PPBP	5
BP	GO:0030198	Extracellular matrix organization	8/56	393/18,862	1.96E − 05	0.008300867	0.007306883	FAP/ITGB3/DDR1/GAS2/HAPLN1/GPM6B/NPNT/COL9A3	8
BP	GO:0043062	Extracellular structure organization	8/56	394/18,862	2.00E − 05	0.008300867	0.007306883	FAP/ITGB3/DDR1/GAS2/HAPLN1/GPM6B/NPNT/COL9A3	8
BP	GO:0045229	External encapsulating structure organization	8/56	396/18,862	2.07E − 05	0.008300867	0.007306883	FAP/ITGB3/DDR1/GAS2/HAPLN1/GPM6B/NPNT/COL9A3	8
BP	GO:0002576	Platelet degranulation	5/56	128/18,862	3.85E − 05	0.011584526	0.010197343	PF4/ITGB3/SELP/MMRN1/PPBP	5
BP	GO:0010743	Regulation of macrophage‐derived foam cell differentiation	3/56	32/18,862	0.000115663	0.027828544	0.024496229	PLA2G2A/PF4/ITGB3	3
MF	GO:0030246	Carbohydrate binding	6/56	267/18,337	0.000158912	0.033371421	0.028436799	CLEC4F/SELP/CHI3L2/ITLN1/CHODL/CLEC1A	6
BP	GO:0010742	Macrophage‐derived foam cell differentiation	3/56	38/18,862	0.000194254	0.03338386	0.029386326	PLA2G2A/PF4/ITGB3	3
BP	GO:0090077	Foam cell differentiation	3/56	38/18,862	0.000194254	0.03338386	0.029386326	PLA2G2A/PF4/ITGB3	3
MF	GO:0048018	Receptor ligand activity	7/56	486/18,337	0.000663852	0.04995539	0.042568502	SEMA6D/CHGB/PF4/GDF3/CMTM5/PPBP/AMH	7
MF	GO:0030546	Signaling receptor activator activity	7/56	492/18,337	0.000713648	0.04995539	0.042568502	SEMA6D/CHGB/PF4/GDF3/CMTM5/PPBP/AMH	7

Abbreviations: AMH, anti‐Müllerian hormone; BP, biological process; CC, cellular component; GO, Gene Ontology; MF, molecular function.

**Table 2:** KEGG enrichment results of differentially expressed genes (DEGs).

ID	Description	GeneRatio	BgRatio	*p* Value	p. adjust	*q* Value	Gene ID	Count
hsa04512	ECM‐receptor interaction	3/24	88/8112	0.002117487	0.168190851	0.142071544	ITGB3/NPNT/COL9A3	3
hsa04062	Chemokine signaling pathway	4/24	192/8112	0.002229456	0.168190851	0.142071544	GNG11/PF4/PLCB4/PPBP	4
hsa04724	Glutamatergic synapse	3/24	114/8112	0.004412108	0.168190851	0.142071544	GNG11/PLCB4/HOMER2	3
hsa05143	African trypanosomiasis	2/24	37/8112	0.00524518	0.168190851	0.142071544	HPR/PLCB4	2
hsa04380	Osteoclast differentiation	3/24	128/8112	0.006096209	0.168190851	0.142071544	FOSB/JUN/ITGB3	3
hsa04926	Relaxin signaling pathway	3/24	129/8112	0.006229291	0.168190851	0.142071544	GNG11/JUN/PLCB4	3
hsa05418	Fluid shear stress and atherosclerosis	3/24	139/8112	0.007656811	0.177200475	0.149682013	JUN/ITGB3/GSTT1	3
hsa05030	Cocaine addiction	2/24	49/8112	0.009063848	0.183542927	0.15503951	FOSB/JUN	2
hsa04060	Cytokine–cytokine receptor interaction	4/24	295/8112	0.010243196	0.184377525	0.155744498	PF4/GDF3/PPBP/AMH	4
hsa05031	Amphetamine addiction	2/24	69/8112	0.017443525	0.20937408	0.176859197	FOSB/JUN	2
hsa05204	Chemical carcinogenesis—DNA adducts	2/24	69/8112	0.017443525	0.20937408	0.176859197	CYP3A5/GSTT1	2
hsa04613	Neutrophil extracellular trap formation	3/24	190/8112	0.017824579	0.20937408	0.176859197	ITGB3/PLCB4/SELP	3
hsa00982	Drug metabolism—cytochrome P450	2/24	72/8112	0.018903018	0.20937408	0.176859197	CYP3A5/GSTT1	2
hsa04151	PI3K‐Akt signaling pathway	4/24	354/8112	0.018961071	0.20937408	0.176859197	GNG11/ITGB3/IRS1/COL9A3	4
hsa04510	Focal adhesion	3/24	201/8112	0.020678105	0.20937408	0.176859197	JUN/ITGB3/COL9A3	3
hsa04971	Gastric acid secretion	2/24	76/8112	0.020926768	0.20937408	0.176859197	KCNJ16/PLCB4	2
hsa00980	Metabolism of xenobiotics by cytochrome P450	2/24	78/8112	0.021971354	0.20937408	0.176859197	CYP3A5/GSTT1	2
hsa05417	Lipid and atherosclerosis	3/24	215/8112	0.024657199	0.221914792	0.187452391	JUN/PLCB4/SELP	3
hsa05163	Human cytomegalovirus infection	3/24	225/8112	0.027738408	0.224957902	0.190022919	GNG11/ITGB3/PLCB4	3
hsa04912	GnRH signaling pathway	2/24	93/8112	0.030471124	0.224957902	0.190022919	JUN/PLCB4	2
hsa04657	IL‐17 signaling pathway	2/24	94/8112	0.03107796	0.224957902	0.190022919	FOSB/JUN	2
hsa04713	Circadian entrainment	2/24	97/8112	0.032927398	0.224957902	0.190022919	GNG11/PLCB4	2
hsa04061	Viral protein interaction with cytokine and cytokine receptor	2/24	100/8112	0.034819573	0.224957902	0.190022919	PF4/PPBP	2
hsa04933	AGE‐RAGE signaling pathway in diabetic complications	2/24	100/8112	0.034819573	0.224957902	0.190022919	JUN/PLCB4	2
hsa04972	Pancreatic secretion	2/24	102/8112	0.036104355	0.224957902	0.190022919	PLA2G2A/PLCB4	2
hsa05142	Chagas disease	2/24	102/8112	0.036104355	0.224957902	0.190022919	JUN/PLCB4	2
hsa04725	Cholinergic synapse	2/24	113/8112	0.043492748	0.249038491	0.210363897	GNG11/PLCB4	2
hsa04726	Serotonergic synapse	2/24	115/8112	0.044892725	0.249038491	0.210363897	GNG11/PLCB4	2
hsa04722	Neurotrophin signaling pathway	2/24	119/8112	0.047742973	0.249038491	0.210363897	JUN/IRS1	2
hsa04935	Growth hormone synthesis, secretion, and action	2/24	119/8112	0.047742973	0.249038491	0.210363897	PLCB4/IRS1	2
hsa00450	Selenocompound metabolism	1/24	17/8112	0.04917022	0.249038491	0.210363897	CTH	1
hsa04919	Thyroid hormone signaling pathway	2/24	121/8112	0.049192788	0.249038491	0.210363897	ITGB3/PLCB4	2

Abbreviations: AGE, Advanced Glycation End; Akt, Ak strain transforming; AMH, anti‐Müllerian hormone; ECM, extracellular matrix; GnRH, Gonadotropin‐Releasing Hormone; IL‐17, Interleukin 17; KEGG, Kyoto Encyclopedia of Genes and Genomes; PI3K, phosphoinositide‐3‐kinase; RAGE, Receptor for Advanced Glycation End.

**Table 3:** GSEA enrichment results of differentially expressed genes (DEGs).

ID	Description	setSize	enrichmentScore	NES	*p* Value	p.adjust	*q* Values	Rank	leading_edge	core_enrichment
hsa04740	Olfactory transduction	379	0.439200854	2.345002562	1.00E − 10	2.00E − 08	1.55E − 08	3563	tags = 35%, list = 21%, signal=28%	OR10A4/OR52E8/OR10H2/OR1Q1/OR1S1/OR2W3/SLC24A4/OR9Q2/PDE2A/OR1E1/OR1J2/OR5AP2/OR10G8/OR10AD1/OR4X2/OR51T1/OR10G3/OR6M1/OR4X1/OR2G6/OR4C3/OR9A2/OR10A2/OR2A5/OR5C1/OR1L4/OR51F1/OR2T1/OR51E2/OR52B2/OR52K2/PRKACG/OR2T4/OR7D2/OR2C1/OR2Y1/OR5F1/OR2F1/OR2A2/GNG13/OR10A6/OR5H6/OR9A4/OR6T1/OR10H1/SLC8A3/OR4K14/OR10G2/OR4A47/OR2M7/OR56A3/OR8J1/OR2J3/ANO2/OR1A1/OR11L1/OR2M3/OR2A25/OR4D11/OR2L2/OR4S1/OR5V1/OR2T33/OR2AE1/OR5D13/OR10G4/OR5K3/OR1L3/OR2T27/OR2AT4/OR7A5/OR2B11/OR5L2/OR52N5/OR8D1/OR8H2/OR51E1/OR8A1/OR6C74/OR4E2/OR52N2/OR2B6/OR14A16/OR10J5/OR11A1/OR1D2/OR51D1/OR6Q1/OR4M1/OR5M3/OR2H1/OR51I2/OR56A4/OR52R1/OR8D2/OR13J1/OR4F29/OR1N2/ARRB2/OR7D4/OR13C4/OR51A7/OR52I2/CNGA4/OR13H1/OR11H6/OR10W1/CALML6/CALML4/OR2B2/OR2L8/OR4D2/OR2K2/OR5D16/OR1L6/OR2AG1/OR4A15/OR2H2/OR8K5/OR5A1/OR10H4/OR9Q1/OR1L8/OR10Z1/ADCY3/OR5B21/OR3A3/OR2S2/CAMK2G/OR1K1/OR2T5/OR56A5/GNAL
hsa04080	Neuroactive ligand‐receptor interaction	335	0.365362389	1.939664425	1.21E − 10	2.00E − 08	1.55E − 08	2298	tags = 29%, list = 14%, signal = 26%	INSL3/UTS2/GABRP/GHRL/S1PR4/P2RY14/CCK/CTSG/GABRR1/PTGIR/P2RX1/S1PR5/GABRA3/KISS1/DRD3/GRIN3B/P2RX2/ADRB3/GHSR/GCG/GIPR/APLNR/SSTR3/CHRM1/CHRM5/GPR156/P2RY6/TACR2/HRH2/CHRND/HCRT/SST/PRLHR/CYSLTR1/PLG/THRB/PTH2/F2RL1/CHRNB4/LPAR2/GABRQ/GRM5/OPRD1/F2RL3/PRSS1/UTS2R/GLP2R/F2RL2/PTAFR/GABRR3/KISS1R/MLN/C3/LEP/GLRA3/MC4R/TRHR/CHRNA10/NTSR1/LYNX1/ADRA1A/CHRNA2/HRH4/GALR2/TAAR5/LHB/NTSR2/CRHR2/HCRTR1/HTR1B/NPW/IAPP/TBXA2R/ADORA1/SSTR5/LTB4R2/GRID2/GLRB/PYY/OXT/P2RY10/FPR1/OPRL1/GRM3/P2RY1/PTGDR/TSHB/GRIK4/GRIA1/ADRA1B/POMC/PENK/VIPR1/HRH3/GABRR2/NMUR1/TACR1
hsa04640	Hematopoietic cell lineage	92	0.49771332	2.158452888	1.39E − 07	1.53E − 05	1.18E − 05	2857	tags = 43%, list = 17%, signal=36%	ITGB3/ITGA2B/IL4/IL3RA/CD1A/GP9/CD1C/IL4R/HLA‐DQA2/CD19/IL2RA/IL6R/IL5RA/CR1L/FLT3/MME/TNF/CD5/CD7/CD1D/GYPA/CSF2RA/IL1R2/GP1BA/CD37/IL9R/CD38/FLT3LG/HLA‐DOB/HLA‐DQA1/CD33/CD1B/CD34/CD1E/CR1/ITGAM/HLA‐DOA/CSF3/IL5/CR2
hsa04060	Cytokine–cytokine receptor interaction	267	0.336098595	1.745217378	7.52E − 07	6.23E − 05	4.81E − 05	3011	tags = 37%, list = 18%, signal = 31%	PPBP/PF4/IL13RA2/PF4V1/LTB/TNFSF14/TNFRSF8/IL36B/IL27/IL4/IL3RA/TNFRSF4/BMP15/CXCR5/IL21R/CD40LG/IL18R1/LTA/BMP10/TNFRSF9/IL4R/GDF5/TNFRSF17/IL2RA/CCL21/IL6R/IL5RA/IL17RB/IL27RA/RELT/CD27/MPL/FASLG/CCR3/CXCL5/NGFR/TNF/CD70/IFNB1/CXCR3/CCL5/CRLF2/CSF2RA/GDF7/TNFSF13B/CCL17/IL1R2/LEP/CCL23/CCR2/TNFSF10/INHBE/IL1RL1/CXCR1/GDF10/IL17B/IL15/IL16/IL21/CXCL12/IL9R/XCR1/IL15RA/TNFRSF18/CX3CL1/CXCR2/CCL24/IL12RB1/IL1RAP/IL34/CCR5/IFNW1/IL26/TNFRSF14/CCL16/TNFRSF25/TNFRSF1B/TNFRSF13C/IL17RE/CCL22/EDA/CCL1/IL1RL2/TNFRSF1A/TNFRSF10A/BMP8B/INHBC/CSF3/NGF/IL5/IL23A/IL17RA/CCR10/PRL/CCR9/TGFBR2/BMP6/CCR4
hsa04390	Hippo signaling pathway	152	−0.4625346	−1.817358277	5.08E − 06	0.000336435	0.000259989	2432	tags = 34%, list = 14%, signal = 29%	APC2/YWHAG/CCND1/PARD3/BMP7/BMP4/FZD8/YWHAQ/WWTR1/GDF6/CRB1/NF2/SMAD2/WNT11/BTRC/SCRIB/PRKCI/CTNNA1/ACTG1/BIRC5/TEAD4/AREG/FZD10/WNT16/YAP1/TEAD1/DLG5/SAV1/PPP2CB/FRMD6/BMP5/ID1/BMPR2/CCND2/TEAD2/AFP/WTIP/TCF7L1/SERPINE1/BMPR1B/FZD3/FZD4/PARD6G/FZD5/WNT5A/AMH/FZD7/FZD6/SMAD1/AJUBA/CDH1
hsa04512	ECM‐receptor interaction	86	−0.520078708	−1.902597121	6.22E − 06	0.000342895	0.000264982	2295	tags = 42%, list = 14%, signal = 36%	ITGAV/COL6A6/SDC1/ITGA2/ITGB6/ITGB1/COL6A1/ITGA9/COMP/ITGB5/VTN/TNN/THBS4/ITGA7/SDC4/LAMB3/COL6A3/SV2A/HSPG2/COL4A1/ITGA6/COL4A2/LAMB2/ITGB8/SPP1/COL2A1/LAMA3/DAG1/ITGA3/COL4A5/LAMC1/COL4A4/LAMA1/LAMA2/COL4A3/NPNT
hsa04613	Neutrophil extracellular trap formation	104	0.42090266	1.85885574	2.01E − 05	0.000951393	0.000735216	2901	tags = 37%, list = 17%, signal = 30%	ITGB3/SELP/CAMP/ITGA2B/MPO/CTSG/ELANE/PADI4/AZU1/AGER/PLCG2/PIK3CD/CR1L/PLCB2/TLR8/ITGB2/SELPLG/CYBA/NCF4/MAPK14/C3/GP1BA/MAPK1/SLC25A31/CLEC7A/SYK/FPR1/ITGAL/SIGLEC9/CR1/HDAC4/AKT1/ITGAM/PRKCB/FGA/NCF2/RAC2/CASP1
hsa04672	Intestinal immune network for IgA production	44	0.5357553	2.018212271	7.14E − 05	0.002726662	0.002107104	3189	tags = 43%, list = 19%, signal = 35%	IL4/CD40LG/TNFRSF17/HLA‐DQA2/MADCAM1/TNFSF13B/ITGB7/ICOSLG/IL15/CXCL12/IL15RA/HLA‐DOB/HLA‐DQA1/TNFRSF13C/HLA‐DOA/IL5/CCR10/CCR9/CCL28
hsa04659	Th17 cell differentiation	106	0.39646178	1.763724481	8.02E − 05	0.002726662	0.002107104	3881	tags = 43%, list = 23%, signal = 34%	IL27/IL4/IL21R/TBX21/IL4R/HLA‐DQA2/IL2RA/IL6R/JAK3/IL27RA/NFATC1/MAPK14/NFATC2/MAPK1/LCK/IRF4/IL21/RARA/IL12RB1/STAT5B/STAT5A/IL1RAP/HLA‐DOB/HLA‐DQA1/ZAP70/HLA‐DOA/IKBKG/LAT/IL23A/STAT1/TGFBR2/MAPK10/RORC/EBI3/MAPK13/IFNGR2/IL17A/IKBKB/FOXP3/STAT6/GATA3/HLA‐DMB/IL17F/CD247/TYK2/RXRG
hsa04662	B cell receptor signaling pathway	79	0.441103001	1.889294856	9.65E − 05	0.002726662	0.002107104	3090	tags = 41%, list = 18%, signal = 33%	LILRA3/LILRA4/CD79B/CD19/PLCG2/PIK3CD/VAV1/NFATC1/DAPP1/LILRA1/CD79A/IFITM1/LILRB3/NFATC2/MAPK1/PTPN6/SYK/LILRA2/LILRB1/INPP5D/LILRB2/BTK/AKT1/PRKCB/LILRA5/IKBKG/RAC2/CR2/LILRB4/CD72/CARD11/FCGR2B
hsa04650	Natural killer cell‐mediated cytotoxicity	103	0.389386846	1.722778561	0.000100364	0.002726662	0.002107104	2922	tags = 35%, list = 17%, signal = 29%	KIR2DS2/KIR3DL2/NCR2/NCR1/PLCG2/PIK3CD/KLRC3/NCR3/VAV1/NFATC1/FASLG/TNF/IFNB1/ICAM2/KLRC1/SH2D1B/ITGB2/NFATC2/SH3BP2/MAPK1/LCK/PTPN6/TNFSF10/HCST/SYK/ITGAL/CD244/ZAP70/TNFRSF10A/PRKCB/MICB/LAT/TYROBP/RAC2/HLA‐C/BID
hsa00280	Valine, leucine, and isoleucine degradation	46	−0.576333657	−1.898329516	0.000100492	0.002726662	0.002107104	5266	tags = 63%, list = 31%, signal = 43%	ACAD8/ECHS1/HIBCH/HSD17B10/ACADSB/AUH/HADHB/PCCA/HADH/ACAT2/DBT/IVD/MCCC1/OXCT1/BCAT2/BCAT1/ACADM/PCCB/ACAT1/EHHADH/HIBADH/AACS/MCCC2/ALDH3A2/HMGCS1/ALDH6A1/BCKDHB/HMGCS2/ALDH7A1
hsa04510	Focal adhesion	195	−0.40849201	−1.657334179	0.000107089	0.002726662	0.002107104	2731	tags = 29%, list = 16%, signal = 25%	TNXB/PIP5K1A/MYLK4/LAMC3/EGFR/ITGAV/COL6A6/ITGA2/ITGB6/ITGB1/COL6A1/MYL9/ITGA9/COMP/ARHGAP35/ITGB5/ACTG1/VTN/TNN/THBS4/ITGA7/MYLK3/CAV1/LAMB3/COL6A3/MET/PDGFD/SHC4/FLNB/HGF/VEGFC/PRKCA/COL4A1/ITGA6/COL4A2/LAMB2/ITGB8/PGF/VCL/CCND2/SPP1/PDGFC/DOCK1/SHC2/PIK3R1/COL2A1/LAMA3/ITGA3/COL4A5/LAMC1/COL4A4/CAV2/LAMA1/PARVA/LAMA2/JUN/COL4A3
hsa05204	Chemical carcinogenesis—DNA adducts	45	−0.574065887	−1.887279155	0.000148862	0.003519518	0.002719806	2165	tags = 33%, list = 13%, signal = 29%	CYP2E1/HSD11B1/GSTO2/EPHX1/CYP3A43/CYP2C18/MGST1/GSTA4/GSTM3/CYP3A4/GSTA2/CYP2C8/GSTA5/CYP3A5/GSTT1
hsa04360	Axon guidance	179	−0.405767112	−1.624656867	0.00018193	0.004014584	0.003102382	2876	tags = 27%, list = 17%, signal = 22%	RASA1/RHOD/PARD3/BMP7/ROBO1/ABL1/NTNG1/PPP3CB/SRGAP1/ITGB1/MYL9/EFNB2/CFL2/NRAS/EFNA3/EFNB1/PLXNB3/SEMA3B/SRGAP3/MET/TRPC3/SMO/ENAH/PRKCA/BMPR2/EFNB3/UNC5B/UNC5C/PIK3R1/EPHA2/RGMA/TRPC1/CAMK2B/BMPR1B/ARHGEF12/NFATC4/PLXNB1/SEMA6A/FZD3/SEMA5A/NRP1/GNAI1/SEMA3C/PARD6G/NTN4/WNT5A/SEMA3A/SEMA6D
hsa00100	Steroid biosynthesis	18	−0.702817327	−1.870009816	0.000217223	0.00426182	0.00329344	3555	tags = 67%, list = 21%, signal = 53%	DHCR7/NSDHL/FDFT1/EBP/LIPA/TM7SF2/LSS/CYP51A1/SQLE/DHCR24/CYP27B1/MSMO1
hsa05340	Primary immunodeficiency	36	0.554430005	1.971700445	0.000218885	0.00426182	0.00329344	3458	tags = 44%, list = 21%, signal = 35%	CIITA/CD40LG/CD19/JAK3/TAP2/CD79A/IGLL1/ORAI1/LCK/BTK/TNFRSF13C/ZAP70/IKBKG/PTPRC/TAP1/DCLRE1C
hsa00982	Drug metabolism—cytochrome P450	50	−0.54728166	−1.827175319	0.000338908	0.006232143	0.004816063	992	tags = 22%, list = 6%, signal = 21%	MGST1/GSTA4/GSTM3/CYP3A4/MAOA/MAOB/GSTA2/CYP2C8/GSTA5/CYP3A5/GSTT1
hsa05310	Asthma	28	0.570927932	1.925361842	0.000527377	0.008856918	0.006844431	2758	tags = 39%, list = 16%, signal = 33%	FCER1A/IL4/CD40LG/HLA‐DQA2/TNF/RNASE3/MS4A2/HLA‐DOB/HLA‐DQA1/HLA‐DOA/IL5
hsa05222	Small‐cell lung cancer	92	−0.462743709	−1.708266317	0.000535161	0.008856918	0.006844431	3967	tags = 40%, list = 24%, signal = 31%	PIK3R3/COL4A6/AKT3/CDK4/GADD45A/CKS2/FN1/POLK/CDK2/CCND1/RB1/LAMC3/ITGAV/ITGA2/ITGB1/CKS1B/CDKN2B/E2F1/TP53/TRAF4/CDK6/LAMB3/CYCS/COL4A1/ITGA6/COL4A2/LAMB2/PIK3R1/LAMA3/ITGA3/COL4A5/LAMC1/COL4A4/LAMA1/LAMA2/NOS2/COL4A3
hsa04380	Osteoclast differentiation	122	0.348860124	1.610550446	0.000586798	0.00924906	0.007147469	3480	tags = 38%, list = 21%, signal = 30%	ITGB3/LILRA3/LILRA4/MAP2K6/PLCG2/PIK3CD/OSCAR/SOCS1/NFATC1/SIRPG/SPI1/LILRA1/TNF/IFNB1/SIRPA/LILRB3/CYBA/NCF4/MAPK14/NFATC2/MAPK1/LCK/SYK/LILRA2/LILRB1/LILRB2/BTK/AKT1/RELB/TNFRSF1A/LILRA5/NCF2/IKBKG/CALCR/TYROBP/STAT1/LILRB4/NFKB2/TGFBR2/MAPK10/FCGR2B/CAMK4/MAPK13/IRF9/TRAF2/IFNGR2
hsa00480	Glutathione metabolism	54	−0.520840532	−1.767790414	0.000647089	0.009735746	0.007523569	1344	tags = 22%, list = 8%, signal = 21%	SMS/CHAC1/ODC1/GPX2/MGST1/GSTA4/LANCL1/GPX8/GSTM3/GSTA2/GSTA5/GSTT1
hsa04350	TGF‐beta signaling pathway	91	−0.461692434	−1.70301083	0.000717845	0.010330719	0.007983351	4456	tags = 46%, list = 27%, signal = 34%	TGFB2/TGFB3/PPP2CA/PPP2R1A/ACVR1B/THBS1/IFNG/BMPR1A/PPP2R1B/ACVR1/ID4/TGIF2/BMP7/E2F5/BMP4/ACVR2A/GDF6/HAMP/NODAL/SMAD2/CDKN2B/INHBB/INHBA/SMAD9/LTBP1/LEFTY2/PPP2CB/FBN1/BMP5/ID1/THSD4/ZFYVE9/BMPR2/SMAD6/ACVR2B/AMHR2/RGMA/FST/BMPR1B/BAMBI/AMH/SMAD1
hsa04310	Wnt signaling pathway	160	−0.394233647	−1.561221525	0.000837823	0.011172891	0.008634163	2580	tags = 25%, list = 15%, signal = 21%	LRP6/FRZB/FZD8/PPP3CB/PRICKLE1/CBY1/WNT11/BTRC/VANGL2/TP53/MMP7/PORCN/FZD10/PLCB3/DKK1/WNT16/PRKCA/CCND2/LRP5/TCF7L1/LGR4/DAAM1/CAMK2B/RSPO4/NFATC4/ZNRF3/ROR1/BAMBI/RNF43/SFRP4/FZD3/FZD4/FZD5/WNT5A/WIF1/FZD7/JUN/FZD6/PLCB4/SFRP5
hsa04062	Chemokine signaling pathway	179	0.312226292	1.524453782	0.000865794	0.011172891	0.008634163	3011	tags = 32%, list = 18%, signal = 27%	PPBP/PF4/GNG11/PF4V1/CXCR5/ADCY4/PIK3R5/GNGT2/CCL21/JAK3/PLCG2/PIK3CD/PIK3R6/VAV1/CCR3/CXCL5/PRKACG/CXCR3/PLCB2/GNG13/CCL5/RASGRP2/CCL17/MAPK1/WAS/CCL23/CCR2/CXCR1/FGR/CXCL12/XCR1/GNG8/CX3CL1/CXCR2/PREX1/CCL24/STAT5B/GNB3/PXN/CCR5/GRK1/CCL16/HCK/ADCY7/CCL22/CCL1/AKT1/ADCY1/FOXO3/PRKCB/IKBKG/CCR10/RAC2/ARRB2/STAT1/CCR9/GRK6/CCR4
hsa04061	Viral protein interaction with cytokine and cytokine receptor	89	0.388551237	1.684197862	0.000877629	0.011172891	0.008634163	2580	tags = 38%, list = 15%, signal = 33%	PPBP/PF4/PF4V1/TNFSF14/CXCR5/IL18R1/LTA/IL2RA/CCL21/IL6R/CCR3/CXCL5/TNF/CXCR3/CCL5/CCL17/CCL23/CCR2/TNFSF10/CXCR1/CXCL12/XCR1/CX3CL1/CXCR2/CCL24/IL34/CCR5/TNFRSF14/CCL16/TNFRSF1B/CCL22/CCL1/TNFRSF1A/TNFRSF10A
hsa04520	Adherens junction	63	−0.492001984	−1.712060802	0.001099441	0.013478326	0.010415752	1934	tags = 29%, list = 12%, signal = 25%	CTNNA1/LMO7/YES1/PTPRF/FARP2/ACTG1/FER/WASL/MET/SORBS1/VCL/TCF7L1/WASF1/TJP1/WASF3/INSR/PTPRM/CDH1
hsa03040	Spliceosome	120	−0.417113025	−1.586037129	0.001418309	0.016766443	0.012956736	6946	tags = 59%, list = 41%, signal = 35%	SNRNP200/CTNNBL1/PRPF38A/THOC1/PCBP1/U2AF1L4/PUF60/USP39/MAGOH/DHX38/LSM2/PRPF6/HNRNPM/LSM4/SNRPB/NCBP2/SNRPB2/RBM17/TXNL4A/SNRPA/SF3B3/TRA2A/CDC5L/HSPA2/HNRNPU/SRSF5/RP9/RBM8A/AQR/EFTUD2/SRSF1/SNRPE/BUD31/CHERP/PRPF31/SNRPG/PRPF38B/THOC2/SRSF3/RBM22/NCBP1/SRSF2/PLRG1/SRSF9/TRA2B/HNRNPA3/SNRPD1/SRSF6/PPIL1/DHX15/BCAS2/PRPF19/PPIE/HNRNPA1L2/SNRNP27/PRPF40B/SNRPF/LSM5/DDX5/SRSF10/DDX42/HSPA8/EIF4A3/WBP11/SRSF7/U2SURP/TCERG1/MAGOHB/SNRPA1/FUS/HSPA1B
hsa04950	Maturity‐onset diabetes of the young	24	0.561866126	1.82758225	0.001719013	0.019620455	0.015162253	1918	tags = 38%, list = 11%, signal = 33%	HHEX/NEUROG3/PKLR/FOXA2/PAX4/BHLHA15/IAPP/HNF1B/FOXA3
hsa04658	Th1 and Th2 cell differentiation	90	0.35578878	1.544338398	0.001854544	0.020461804	0.015812429	3887	tags = 42%, list = 23%, signal = 33%	IL4/TBX21/IL4R/HLA‐DQA2/IL2RA/JAK3/RUNX3/NFATC1/DLL4/MAPK14/NFATC2/MAPK1/LCK/IL12RB1/STAT5B/STAT5A/HLA‐DOB/HLA‐DQA1/NOTCH1/ZAP70/HLA‐DOA/IKBKG/LAT/IL5/MAML3/STAT1/MAPK10/RBPJL/MAPK13/IFNGR2/MAML1/IKBKB/STAT6/GATA3/HLA‐DMB/CD247/TYK2/STAT4
hsa04110	Cell cycle	124	−0.408048192	−1.565505467	0.001933244	0.020642059	0.015951726	5544	tags = 47%, list = 33%, signal = 32%	SMC1A/RBX1/ORC5/ANAPC7/CUL1/CDKN2A/ORC3/YWHAH/HDAC2/CDC7/CCNB3/CDC16/SKP1/PRKDC/SFN/SMC1B/CCNH/CDKN1A/TGFB2/ANAPC4/TGFB3/MCM3/CDC6/MCM2/CDKN1C/CDK4/GADD45A/SMC3/ESPL1/CDKN2C/CDC23/YWHAG/CDK2/CCND1/RB1/ANAPC10/CHEK1/ABL1/E2F5/YWHAQ/MCM4/SMAD2/CDKN2B/E2F1/TTK/TP53/CDK6/ORC6/MCM6/MAD2L1/CDC45/CDC25C/CDC25A/CCND2/BUB1/CCNA1/BUB1B/WEE1
hsa03013	Nucleocytoplasmic transport	92	−0.44162622	−1.630308918	0.002139202	0.02212737	0.017099542	6266	tags = 58%, list = 37%, signal = 36%	NCBP2/NXT2/THOC6/NUP88/SUMO3/NUP37/RANBP2/DDX19B/RBM8A/NUP35/SENP2/KPNA6/EEF1A1/THOC2/XPO5/NUP210L/DDX19A/NCBP1/THOC7/NUP43/RANGAP1/IPO9/NUP205/NUP54/TNPO1/CSE1L/UPF3B/NUP93/AAAS/XPOT/SEH1L/NUP210/PHAX/RNPS1/IPO7/SNUPN/KPNA5/TNPO2/NMD3/RAN/NUP188/PNN/NUP107/XPO4/EIF4A3/NUP160/IPO5/NUP155/MAGOHB/XPO1/RANBP17/EEF1A2/KPNA7
hsa05320	Autoimmune thyroid disease	40	0.475368328	1.755634326	0.00225034	0.022571589	0.017442824	3236	tags = 32%, list = 19%, signal = 26%	IL4/CD40LG/HLA‐DQA2/FASLG/CTLA4/HLA‐F/TSHB/HLA‐DOB/HLA‐DQA1/HLA‐DOA/IL5/HLA‐C/TPO
hsa05100	Bacterial invasion of epithelial cells	68	−0.458449497	−1.617083461	0.002382542	0.023076806	0.017833245	2541	tags = 26%, list = 15%, signal = 23%	CD2AP/ARHGAP10/ITGB1/CTNNA1/ACTG1/CAV1/ARHGEF26/WASL/MET/SHC4/VCL/DOCK1/SHC2/PIK3R1/WASF1/CTTN/CAV2/CDH1
hsa05146	Amebiasis	99	−0.42151303	−1.566767448	0.002440146	0.023076806	0.017833245	1953	tags = 25%, list = 12%, signal = 22%	HSPB1/ARG2/LAMB3/IL10/PLCB3/PRDX1/CXCL2/PRKCA/COL4A1/COL4A2/LAMB2/VCL/SERPINB13/PIK3R1/LAMA3/COL4A5/LAMC1/COL4A4/LAMA1/SERPINB3/LAMA2/NOS2/COL4A3/PLCB4/SERPINB4
hsa04392	Hippo signaling pathway—multiple species	27	−0.59822154	−1.738962278	0.002768258	0.025082014	0.019382826	2342	tags = 44%, list = 14%, signal = 38%	WWTR1/NF2/FAT4/TEAD4/YAP1/TEAD1/SAV1/FRMD6/TEAD2/WTIP/DCHS1/AJUBA
hsa04141	Protein processing in endoplasmic reticulum	157	−0.383089525	−1.510497474	0.00280373	0.025082014	0.019382826	6245	tags = 55%, list = 37%, signal = 35%	EDEM1/SEC. 61A2/DNAJA1/BAG2/SSR1/EIF2AK4/AMFR/SYVN1/UBE2D4/LMAN2/HSPA2/SAR1A/PLAA/UBE4B/RBX1/RRBP1/CUL1/VCP/UBQLN1/CAPN2/SSR2/DNAJC1/SEC. 24B/ATF4/SEC. 24 C/UBQLN4/SKP1/MAPK8/RAD23A/SIL1/PRKCSH/SEC. 24D/DDOST/HYOU1/CALR/SEC. 61B/SEC. 24 A/MAN1A2/EIF2S1/HSPBP1/SEC. 62/DNAJC10/DDIT3/RPN2/TRAM1/ERLEC1/XBP1/SEC. 61 G/SVIP/DERL1/DNAJB11/DNAJB1/P4HB/UBXN4/HSP90B1/MBTPS1/HSPA8/PDIA4/UGGT2/SEC. 23B/HERPUD1/LMAN1/HSPH1/SAR1B/HSP90AB1/SEL1L2/HSP90AA1/UBXN2A/SEC. 61A1/SSR3/SEC. 23 A/GANAB/MBTPS2/PPP1R15A/SEC. 31 A/EIF2AK3/HSPA4L/STT3A/HSPA5/MAP3K5/PDIA6/SEC. 63/UBXN8/TUSC3/WFS1/HSPA1B/CRYAB
hsa05330	Allograft rejection	31	0.527004266	1.816580628	0.002939245	0.025602371	0.019784945	2849	tags = 35%, list = 17%, signal = 30%	IL4/CD40LG/HLA‐DQA2/FASLG/TNF/HLA‐F/HLA‐DOB/HLA‐DQA1/HLA‐DOA/IL5/HLA‐C
hsa00330	Arginine and proline metabolism	44	−0.514587618	−1.678332387	0.004236214	0.035623593	0.027529124	1754	tags = 27%, list = 10%, signal = 24%	ARG2/P4HA2/SMS/ALDH3A2/ODC1/CKMT2/GATM/ALDH7A1/CKB/MAOA/MAOB/NOS2
hsa00270	Cysteine and methionine metabolism	46	−0.491921488	−1.620292464	0.004352636	0.035623593	0.027529124	5339	tags = 59%, list = 32%, signal = 40%	AHCYL2/DNMT3B/GSS/LDHB/MTAP/CDO1/SDSL/MDH2/GOT1/LDHA/AHCYL1/GCLC/BCAT2/MDH1/ADI1/ENOPH1/AHCY/BCAT1/MTR/BHMT2/MAT1A/BHMT/SMS/MAT2A/PHGDH/PSAT1/CTH
hsa00260	Glycine, serine, and threonine metabolism	36	−0.535202886	−1.674685913	0.00441259	0.035623593	0.027529124	3514	tags = 44%, list = 21%, signal = 35%	ALAS1/GRHPR/SRR/AOC3/CHDH/GCAT/BHMT/DMGDH/GATM/PHGDH/ALDH7A1/PSPH/PSAT1/MAOA/MAOB/CTH
hsa05412	Arrhythmogenic right ventricular cardiomyopathy	76	−0.447911817	−1.610959312	0.004852527	0.037360097	0.028871056	2377	tags = 41%, list = 14%, signal = 35%	CACNB3/RYR2/ITGAV/ITGA2/ITGB6/ITGB1/CACNA2D1/CTNNA1/ITGA9/ITGB5/ACTG1/ITGA7/SLC8A2/LMNA/ATP2A2/ITGA6/ITGB8/GJA1/PKP2/CDH2/TCF7L1/SGCG/JUP/DAG1/DSG2/ITGA3/LAMA1/SGCB/DSP/SGCD/LAMA2
hsa05033	Nicotine addiction	40	0.453788687	1.675936213	0.004924933	0.037360097	0.028871056	2761	tags = 28%, list = 16%, signal = 23%	GABRP/GABRR1/GABRA3/GRIN3B/GABRQ/GABRR3/GRIA1/CACNA1B/GABRR2/CACNA1A/GABRG2
hsa00310	Lysine degradation	48	−0.4822858	−1.596379535	0.004966297	0.037360097	0.028871056	5228	tags = 50%, list = 31%, signal = 35%	ECHS1/DOT1L/MECOM/PRDM6/SMYD3/SETD2/SETDB2/HADH/ACAT2/SMYD2/SETMAR/SUV39H1/SETD7/DLST/ACAT1/PLOD3/EHHADH/SUV39H2/ALDH3A2/PLOD2/BBOX 1/AADAT/ALDH7A1/AASS
hsa04725	Cholinergic synapse	111	0.332006816	1.493292226	0.005387054	0.039624778	0.030621152	2618	tags = 31%, list = 16%, signal = 26%	GNG11/KCNQ3/ADCY4/PIK3R5/GNGT2/CHAT/KCNJ18/ITPR3/PIK3CD/CHRM1/CHRM5/PIK3R6/KCNJ2/CACNA1F/CHRNB4/PRKACG/PLCB2/KCNQ2/GNG13/SLC18A3/MAPK1/CREB3L1/CREB5/GNG8/GNB3/CACNA1B/KCNQ4/GNAO1/KCNJ3/ADCY7/CACNA1A/AKT1/ADCY1/PRKCB
hsa04742	Taste transduction	82	0.359201051	1.524161067	0.00639565	0.046020875	0.035563914	2371	tags = 28%, list = 14%, signal = 24%	CALHM1/GABRA3/HTR3A/ADCY4/TAS1R1/P2RX2/TAS2R41/ITPR3/TAS2R39/PRKACG/PLCB2/GNG13/TAS1R3/TAS2R5/ENTPD2/HTR1B/TRPM5/GNB3/P2RY1/PKD2L1/TAS1R2/HTR3E/CACNA1A
hsa01200	Carbon metabolism	107	−0.398718081	−1.503472278	0.006935893	0.048846394	0.037747412	5743	tags = 49%, list = 34%, signal = 32%	ALDOA/PDHA1/GOT2/SUCLG2/ACOX3/PGAM4/SDHC/ECHS1/HIBCH/ALDOC/CS/PGK1/HK1/PCCA/FH/RPE/SDSL/GLUD2/MDH2/TPI1/IDH1/ACAT2/ADH5/GOT1/ME3/ACO2/ME2/MDH1/GPT2/GPI/GAPDH/ENO2/DLST/ENO1/HAO2/PCCB/ACAT1/ESD/PFKM/ACO1/IDH3A/SUCLA2/DLAT/H6PD/PRPS2/ALDH6A1/PHGDH/ME1/OGDHL/RGN/PSPH/PSAT1

Abbreviations: AMH, anti‐Müllerian hormone; AMHR, receptor AMH; ECM, extracellular matrix; GSEA, gene set enrichment analysis; IgA, Immunoglobulin A; NES, normalized enrichment score; TGF‐beta, Transforming Growth Factor‐beta; Th17, T helper 17 cells.

GO enrichment analysis identified 11 enriched terms, with the five most significant being platelet alpha granule, extracellular matrix organization, extracellular structure organization, external encapsulating structure organization, and platelet degranulation. These terms are predominantly associated with platelet function.

A total of 32 enriched pathways were found in the KEGG enrichment analysis, of which the five most significant pathways were extracellular matrix (ECM)‐receptor interactions, chemokine signaling pathways, the glutamatergic synapse pathway, the African trypanosomiasis pathway, and the osteoclast differentiation pathway. These results echo the GO enrichment results, such as the enrichment of ECM‐related processes.

In the GSEA enrichment analysis, 47 KEGG pathways were found to be enriched. The five most significant pathways were olfactory transduction, neuroactive ligand–receptor interactions, haematopoietic cell lineage, cytokine–cytokine receptor interactions, and the Hippo signaling pathway, which mainly include BPs related to the function of the blood system and the interaction of cell receptors and ligands.

### 3.7 Immune Infiltration Analysis

**Figure 8:**
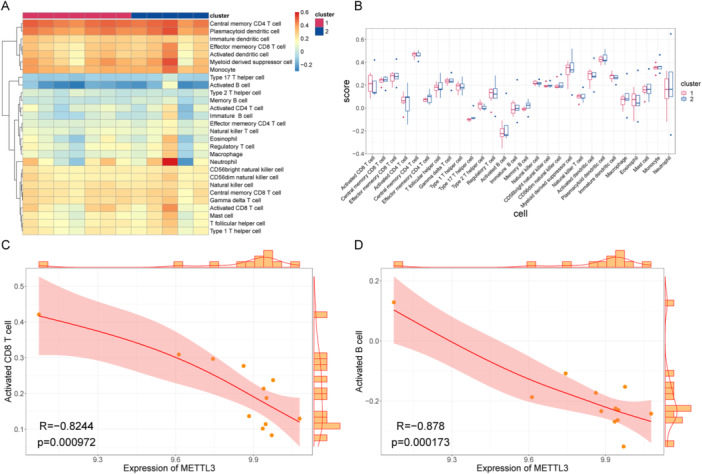
The ssGSEA assessment of immune infiltration. (A) Immunoscore heatmap. Twenty‐eight types of immune cells were listed as samples. (B) Box plot of immunoscore. The *x*‐axis is the 28 types of immune cells, the *y*‐axis is the immune infiltration level, and each color represents a sample group. (C) Correlation scatter plot of METTL3‐activated CD8 T cells. (D) Correlation scatter plot of METTL3‐activated B cells. CD8, cluster of differentiation 8; METTL3, methyltransferase‐like 3; ssGSEA, single‐sample gene set enrichment analysis.

Immune infiltration analysis was conducted by scoring 28 types of immune cells using the ssGSEA method, with heatmaps and box plots employed for visualization(Figure 8A,B). It was found that many of the 28 types of immune cells were significantly different between the two groups, such as activated cluster of differentiation 8 (CD8) T cells, type 17 T helper cells, and type 2 T helper cells. Additionally, correlations between hub genes and immune cell infiltration levels revealed that METTL3 exhibited significantly strong negative correlations with activated CD8 T cells and activated B cells, highlighting its potential research significance (Figure 8C,D).

**Figure 9:**
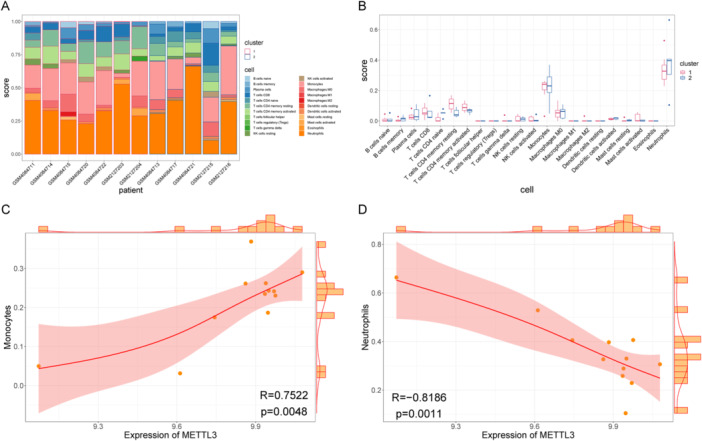
CIBERSORT assessment of immune infiltration. (A) Stacked histogram of immunoscore, (B) box plot of immunoscore, (C) correlation scatter plot of METTL3‐monocytes, and (D) correlation scatter plot of METTL3‐neutrophils. METTL3, methyltransferase‐like 3.

Another method, namely, CIBERSORT, was used to calculate the immune infiltration levels of 22 types of immune cells in the two groups of samples (Figure 9A,B). In addition, we calculated the direct correlation between all hub genes and the levels at which immune cells infiltrated. The CIBERSORT results were consistent with the above analysis results, with METTL3 gene expression being significantly correlated with a variety of immune cells (Figure 9C,D). METTL3 was positively correlated with monocytes and negatively correlated with neutrophils, and these data supplement the ssGSEA results. More importantly, METTL3 is the hub gene with the highest degree, so it has the most extensive regulatory functions. The above findings echo the other results obtained in this study.

## 4 Discussion

PCOS is one of the most prevalent reproductive endocrine disorders in women of childbearing age, characterized by ovarian polycystic changes, ovulation dysfunction, and signs of hyperandrogenism or hyperandrogenaemia. PCOS exhibits a high degree of heterogeneity among females and is closely associated with chronic inflammation and cellular metabolic abnormalities [31]. The process of follicular development in PCOS patients is influenced by alterations in the follicular microenvironment, dysfunction of ovarian GCs, and an enhanced inhibitory effect of anti‐Müllerian hormone (AMH) and its receptor (AMHR) on early follicular development.

In eukaryotic cells, m6A RNA methylation is dynamically regulated by methyltransferases, demethylases, and binding proteins, which collectively influence the growth and development of germ cells. Aberrant m6A modifications can disrupt the balance of key regulatory genes, impairing cellular homeostasis and contributing to disease pathogenesis. Ovarian GCs play a critical role in determining follicular fate and regulating oocyte development and maturation. However, the dynamic regulatory network and biological significance of m6A in GCs remain unclear.

Since Kelly et al. first proposed in 2001 that chronic inflammation may be present in PCOS patients, an increasing body of evidence has demonstrated that immune‐inflammatory mechanisms are intricately linked to the pathogenesis and progression of PCOS [32, 33]. Recent studies have reported elevated numbers of peripheral blood lymphocytes, monocytes, and eosinophils, as well as macrophage and lymphocyte infiltration into ovarian tissues in PCOS patients [34]. Furthermore, peripheral blood and ovarian immune cells, along with increased immune molecular gene polymorphisms, are strongly correlated with PCOS symptoms. Human preovulatory follicles contain various immunoactive cells, including T cells, natural killer (NK) cells, and monocyte‐derived macrophages. Aberrant expression of these immune cells may lead to immune dysfunction or shifts in the proportions of immune‐related secreted factors, ultimately contributing to PCOS pathogenesis [35]. Exploring the dynamic regulatory mechanisms of m6A in ovarian GCs and investigating its correlation with the immune mechanisms of PCOS could provide novel insights for the clinical diagnosis and treatment of PCOS from an epigenetic perspective.

This study represents the first attempt to investigate the role of 36 common m6A RNA methylation regulators in PCOS. It has been well established that the developmental maturation of oocytes is critically dependent on the precise regulation of m6A modifications.

METTL3, highly conserved across eukaryotes from yeast to humans, plays a crucial role in early development [36]. Studies using zebrafish and mouse models demonstrate a developmental arrest in METTL3 mutants and a reduction in egg pole body expulsion in knockout mice [37]. Immunofluorescence analysis showed that METTL3 is localized to the enrichment of mRNA processing factors in the nucleoplasm and nuclear spots, suggesting that it can regulate protein formation by regulating mRNA translation efficiency and can affect reproductive functions, such as gamete formation, by increasing apoptosis. Ovarian GC dysfunction is implicated in PCOS. Comparative analyses of m6A methylation levels in luteinized GCs from normovulatory women and nonobese PCOS patients revealed differences in m6A profiles, with higher levels detected in PCOS patients [38]. Specifically, METTL3, METTL14, FTO, and ALKBH5 expression levels were significantly elevated in PCOS patients compared with controls.

FTO, a gene implicated in obesity and IR [39], is also linked to PCOS. Overweight/obesity and IR are frequently observed in PCOS, and some studies suggest an association between FTO and PCOS‐related insulin resistance (PCOS‐IR). The SNP rs9939609 in the FTO gene has been shown to be significantly associated with PCOS [40, 41]. Studies examining FTO and ALKBH5 expression, along with total m6A content, in human and mouse GCs suggest a potential role for m6A regulation in GCs [42, 43]. Overexpression of FTO promotes GC proliferation, inhibits apoptosis, and induces IR in GCs. Furthermore, FTO‐mediated GC dysfunction may be linked to upregulation of FLOT2 [44]. Rein, a competitive inhibitor of FTO substrates, may represent a novel therapeutic target for obesity and PCOS‐IR by hindering the binding of single‐stranded RNA substrates to FTO active sites [45].

Studies indicate that oocytes with protein defects in the YTH domain exhibit developmental arrest [46, 47]. Deletion of YTHDF1 results in increased selective polyadenylation, disrupting normal mRNA translation and significantly reducing follicular maturation rates. Functional studies of YTHDF2 mutations confirm its critical role in the production of mature oocytes (MII eggs), likely mediated through maternal RNA degradation and modulation of MII transcripts [48, 49].

On the basis of the GSE137684 and GSE80432 data sets, we observed that certain genes exhibited distinct expression patterns among the three m6A functional types. Gene correlation analysis of writers and erasers revealed positive correlations between the following four gene pairs in PCOS patients: METTL3‐ALKBH3, METTL14‐ZC3H13, WTAP‐CBLL1, and CBLL1‐PCIF1. These findings suggest potential synergistic relationships among three pairs of writer genes (METTL14‐ZC3H13, WTAP‐CBLL1, and CBLL1‐PCIF1). Additionally, a negative feedback regulatory mechanism may exist between METTL3 and ALKBH3.

Considering the biological significance of m6A‐regulated genes, we developed a diagnostic model incorporating all 11 m6A genes, which collectively formed the PCOS diagnostic framework. The five genes with the largest absolute effect coefficients were WTAP, METTL14, ZC3H13, PCIF1, and RBM15. Validation of the model's predictive performance using the external data set GSE80432 demonstrated its robustness and superior accuracy. Furthermore, unsupervised clustering of hub genes identified six genes (METTL14, HNRNPA2B1, YTHDF3, YTHDF2, YTHDC1, and YTHDC2) as potential key distinguishing factors.

The 65 DEGs between the two sample groups were subjected to GO, KEGG, and GSEA enrichment analyses. The top five most significant pathways identified by each method were as follows: platelet alpha granule, extracellular matrix organization, extracellular structure organization, external encapsulating structure organization, and platelet degranulation (GO analysis); ECM‐receptor interaction, chemokine signaling pathway, glutamatergic synapse, African trypanosomiasis, and osteoclast differentiation (KEGG analysis); and olfactory transduction, neuroactive ligand–receptor interaction, hematopoietic cell lineage, cytokine–cytokine receptor interaction, and Hippo signaling pathway (GSEA).

Additionally, ssGSEA was employed to compare m6A regulator levels with immune cell infiltration in PCOS. Significant differences were observed in activated CD8 T cells, type 17 T helper cells, type 2 T helper cells, and memory B cells between the two sample groups. METTL3 exhibited a highly significant and strong negative correlation with both activated CD8 T cells and activated B cells. CIBERSORT analysis further indicated that METTL3 expression levels were significantly associated with various immune cells, showing a positive correlation with monocytes and a negative correlation with neutrophils. Notably, METTL3 appears to have extensive regulatory functions in PCOS.

Building upon previous findings, this study investigates the translational potential of m6A regulatory factors in PCOS. Key m6A genes identified, including METTL14 and WTAP, demonstrate altered expression in PCOS patient samples, providing a biological basis for early diagnostic screening and risk stratification. Specifically, the expression profiles of METTL14 and WTAP may serve as candidate biomarkers for molecular PCOS subtyping and personalized diagnostics. Consistent with existing literature, these genes exhibit favorable detectability and specificity across various biological samples, supporting the development of noninvasive or minimally invasive diagnostic approaches.

Beyond diagnostics, therapeutic strategies targeting m6A regulatory factors show considerable promise. Current research demonstrates the ability of various small‐molecule inhibitors or activators to modulate the activity of m6A modification‐related enzymes, including those targeting METTL3 and FTO, which are currently in preclinical or early clinical phases in other disease contexts, such as cancer and metabolic disorders. In PCOS, targeted modulation of m6A methyltransferases (writers) or demethylases (erasers) may potentially influence the ovarian microenvironment, improve GC function, and mitigate chronic inflammation by impacting immune cell infiltration, offering novel therapeutic avenues for personalized PCOS management. However, further validation is required regarding the specificity, safety, and long‐term efficacy of these potential therapies. A deeper understanding of the molecular mechanisms of the m6A regulatory network in PCOS pathogenesis is essential for guiding clinical translation.

In summary, m6A regulatory factors show promise as potential biomarkers for early PCOS diagnosis and subtyping, and provide a framework for innovative therapeutic approaches targeting epigenetic modifications. Future multi‐center, large‐scale clinical studies, coupled with functional experiments and translational research, are crucial for advancing the practical application of m6A‐related molecules in PCOS clinical management.

## 5 Conclusions

This study employed ssGSEA and other advanced multidimensional analytical approaches to systematically investigate the relationship between m6A regulatory factors and immune cell infiltration in PCOS. The findings revealed significant differences in the levels of activated CD8 T cells, Th17 cells, Th2 cells, and memory B cells across different groups. Notably, METTL3 exhibited a highly significant negative correlation with activated CD8 T cells and activated B cells, a positive correlation with monocytes, and a negative correlation with neutrophils, indicating its pivotal role in the regulation of the immune microenvironment in PCOS.

However, it is important to note that this study primarily relied on bioinformatics analyses derived from publicly available multi‐cohort data sets, and lacks validation through in vivo and in vitro experimental studies. Consequently, the conclusions drawn require further substantiation through functional experiments.

To address these limitations, future research will focus on conducting in vivo and in vitro experiments using PCOS patient samples and animal models. Through approaches, such as gene knockout, overexpression, and immunodetection, we aim to comprehensively elucidate the biological functions of key m6A regulatory factors and their causal relationships with immune infiltration. This will provide a more robust foundation for understanding the underlying mechanisms. Additionally, we underscore that the bioinformatics findings presented herein serve as valuable leads for subsequent mechanistic investigations.

## Author Contributions

**Wenting Xu:** conceptualization, data curation, formal analysis, funding acquisition, methodology, writing – original draft. **Aifang Lu:** conceptualization, data curation, methodology, visualization. **Lijuan Cui:** formal analysis, investigation, methodology. **Haiqing Qian:** investigation, methodology, visualization. **Jiahui Wang:** data curation, methodology, visualization. **Mengyu Tang:** investigation, project administration, software. **Lili Zhu:** data curation, methodology, writing – original draft, writing – review and editing. **Lihong Wang:** conceptualization, project administration, writing – review and editing.

## Conflicts of Interest

The authors declare no conflicts of interest.

## Data Availability

The data sets generated and analyzed during the current study are available from GEO, STRING analysis, Xiantao Academic Tools (https://www.xiantaozi.com) that provide free online tools and resources. The authors confirm that the data supporting the findings of this study are available within the article.
